# The Association between Serum Magnesium Levels and Depression in an Adult Primary Care Population

**DOI:** 10.3390/nu11071475

**Published:** 2019-06-28

**Authors:** Emily K. Tarleton, Amanda G. Kennedy, Gail L. Rose, Abigail Crocker, Benjamin Littenberg

**Affiliations:** 1 Office of Clinical Trials Research, Larner College of Medicine, University of Vermont, Burlington, VT 05405, USA; 2 Department of Medicine Quality Program, Larner College of Medicine, University of Vermont, Burlington, VT 05405, USA; 3 Department of Psychiatry, Larner College of Medicine, University of Vermont, Burlington, VT 05405, USA; 4 Department of Mathematics and Statistics, College of Engineering and Mathematics, University of Vermont, Burlington, VT 05405, USA; 5 Department of Medicine, Larner College of Medicine, University of Vermont, Burlington, VT 05405, USA

**Keywords:** magnesium, depression, primary care

## Abstract

Depression is common, places a large burden on the patient, their family and community, and is often difficult to treat. Magnesium supplementation is associated with improved depressive symptoms, but because the mechanism is unknown, it is unclear whether serum magnesium levels act as a biological predictor of the treatment outcome. Therefore, we sought to describe the relationship between serum magnesium and the Patient Health Questionnaire (PHQ, a measure of depression) scores. A cross-sectional analysis of medical records from 3604 adults (mean age 62 years; 42% men) seen in primary care clinics between 2015 and 2018, with at least one completed PHQ were included. The relationship between serum magnesium and depression using univariate analyses showed a significant effect when measured by the PHQ-2 (−0.19 points/mg/dL; 95% CI −0.31, −0.07; *P* = 0.001) and the PHQ-9 (−0.93 points/mg/dL; 95% CI −1.81, −0.06; *P* = 0.037). This relationship was strengthened after adjusting for covariates (age, gender, race, time between serum magnesium and PHQ tests, and presence of diabetes and chronic kidney disease) (PHQ-2: −0.25 points/mg/dL; 95% CI −3.33, −0.09; *P* < 0.001 and PHQ-9: −1.09 95% CI −1.96 −0.21; *P* = 0.015). For adults seen in primary care, lower serum magnesium levels are associated with depressive symptoms, supporting the use of supplemental magnesium as therapy. Serum magnesium may help identify the biological mechanism of depressive symptoms and identify patients likely to respond to magnesium supplementation.

## 1. Introduction

Depression is a common mental health disorder characterized by persistent low mood, lack of energy, and loss of interest or pleasure in things [1]. It impacts quality of life and is a leading contributor to disease burden, based on disability-adjusted life years [2]. In 2016, almost 7% of US adults experienced at least one major depressive episode in the past year [3]. The lifetime prevalence of a mood disorder is almost 30% in primary care populations [4,5] and patients are more likely to receive treatment from their primary care providers than from mental health professionals [5,6]. 

In-office screening in primary care increases the identification of adults with depression and is recommended by the US Preventative Services Task Force [6]. Once depression is identified, however, providers and patients often struggle to find safe, tolerable, rapid, effective, and inexpensive treatment. First-line treatments, such as cognitive behavioral therapy and antidepressant medications, can take weeks to have an effect and often fail to improve symptoms altogether [7]. Biomarkers may help identify the most suitable options, leading to more individualized and tailored treatment plans. However, more data are needed to identify practical and useful biomarkers [8]. 

Supplementation with magnesium has been shown to decrease symptoms of depression in patients with mild to moderate depression [9,10]. As the fourth most abundant mineral and second most abundant cation in the cell, magnesium is a cofactor for over 300 enzymes and is essential for anaerobic and aerobic energy production, glycolysis, mitochondrial oxidative phosphorylation, as well as potassium and calcium regulation [11,12]. Adequate magnesium intake is essential for energy production, prevention of dysrhythmias, blood pressure regulation, avoidance of insulin resistance, and bone homeostasis [13,14]. Inadequate magnesium intake has been associated with several chronic health conditions, including diabetes, cardiovascular disease, and chronic pain [14,15]. 

Hypomagnesaemia is associated with neuromuscular, cardiovascular, neurologic, and electrolyte abnormalities [16,17]. Although low magnesium intake has been associated with depression in several different parts of the world [18,19], serum magnesium levels have been reported as high, normal, and low in depressed patients, and it is not clear whether the magnesium status prior to treatment influences outcomes. Recent review articles report a positive effect of magnesium on symptoms of depression [17,20]; however, there is still some speculation as to the strength of the relationship [21,22]. Interpretation of these studies are limited by lack of information about the distribution of clinical subgroups in the samples, types of treatment, severity of illness at baseline, and magnesium homeostasis [23]. Whether patients suffering from depression have lower serum magnesium levels than those without depression is not clear [24]. Therefore, we sought to clarify the relationship of serum magnesium to depressive symptoms in adults being screened for depression in primary care while controlling for the effects of possibly confounding demographic and clinical factors.

## 2. Materials and Methods

We performed a cross-sectional secondary analysis of medical records of adults seen in any of the 8 primary care clinics of The University of Vermont Medical Center in Chittenden County, Vermont (Burlington and surrounding areas). All data were de-identified and were obtained from medical records via the medical center’s electronic data warehouse. As a health records review study, The University of Vermont Research Protections Office approved a waiver of consent. Eligible subjects were 18 years or older, had at least one serum magnesium level reported by the medical center laboratory, and at least one depression screening score recorded between November 1, 2015 and October 31, 2018. This study was approved by the University of Vermont Committee on Human Research.

The primary predictor was serum magnesium (measured in mg/dL by colorimetric reflectance spectrophotometry; normal range for age >18 years defined as 1.7 to 2.8 mg/dL [25]). The primary outcome was the Patient Health Questionnaire-2 (PHQ-2) score. PHQ-2 is a validated tool for assessing depression and is the short version of a longer questionnaire, PHQ-9 [26]. The two items on the PHQ-2 assess whether patients “feel down, depressed, or hopeless” and are experiencing “little interest or pleasure” in things. The PHQ-2 ranges from 0 to 6; scores of 3 or more are considered positive (sensitivity 61%; specificity 92%) and suggest that the PHQ-9 should be administered to assess symptom severity and possible diagnosis of major depressive disorder [26]. A secondary outcome was the PHQ-9 score. Scores can range from 0 to 27, with the following categories of severity: 0–4 none; 5–9 mild; 10–14 moderate; 15–19 moderate to severe; 20-27 severe. Scores greater than 10 have a sensitivity of 88% and a specificity of 88% for major depression [27]. PHQ-2 was selected as the primary outcome to maximize available sample size and generalizability, as the PHQ-9 is often used only in response to an elevated PHQ-2 score, at the discretion of providers to further evaluate suspected depression or to monitor treatment response. 

Several covariates were considered for the analyses. The time between the serum magnesium test and the PHQ survey was calculated as the absolute difference in their dates and expressed as months. When the subjects had more than one serum magnesium or PHQ survey, we selected the pair that were performed the closest to each other in time. Age at the time of the PHQ survey, to the nearest year, was calculated based on date of birth recorded in the electronic health record. Gender was used as recorded in the electronic health record. Due to the low proportion of non-white patients in the population, race was categorized as white or non-white. Diabetes was considered present if the patient had any visits with the International Classification of Disease 10th edition (ICD10) codes beginning with E08, E09, E10, E11, E13, or O24. Chronic kidney disease (CKD) was considered present if the patient had any visits with ICD10 codes of N18.4, N18.5, N18.6, Z99.2, or T82.4 (CKD stages 4 or 5 or end-stage renal disease). Diagnosis data were not available for 6 patients and, because magnesium status is affected by diabetes and CKD, they were excluded from the multivariable analyses. 

To assess the relationship between serum magnesium and the PHQ-2 score, we performed both unadjusted (univariate) and multiple regression to control for possible confounders. To protect against the possibility that departures from the assumptions of least-squares regression were introducing important errors, we repeated the analyses using Huber-weighted robust linear regression [28], which does not require those assumptions. We calculated the 95% confidence intervals (95% CI) and *P*-values for all estimates. *P*-values <0.05 were considered statistically significant. We repeated the analysis using PHQ-9 as the outcome variable. All calculations were performed in Stata version 15.1 (StataCorp, College Station, TX, USA).

## 3. Results

**Figure 1:**
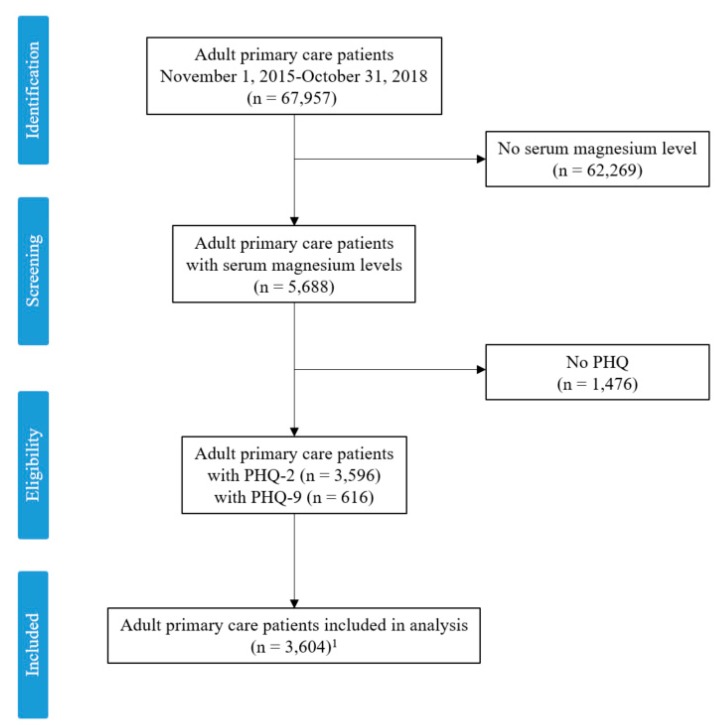
Consort diagram. Patient Health Questionnaire (PHQ); number (n), ^1^ 608 subjects had both a PHQ-2 and PHQ-9, leaving the final sample size of unique patients at 3604.

A total of 67,957 individual adult patients had visits between November 1, 2015 and October 31, 2018. A total of 62,269 patients had no serum magnesium levels recorded, leaving 5688 potentially eligible subjects. Of these, 3596 had at least one PHQ-2 survey recorded and 616 had at least one PHQ-9 survey recorded (Figure 1). A total of 608 subjects had both PHQ-2 and PHQ-9 results available and were included in both analyses. The distribution of PHQ-2 scores reflects a screening population with a mean of 1.0 points (standard deviation = 1.6). The PHQ-9 scores had a mean of 12.6 points (standard deviation = 6.2) and most subjects scored “mild”, “moderate”, or “severe”. See Table 1.

**Figure 2:**
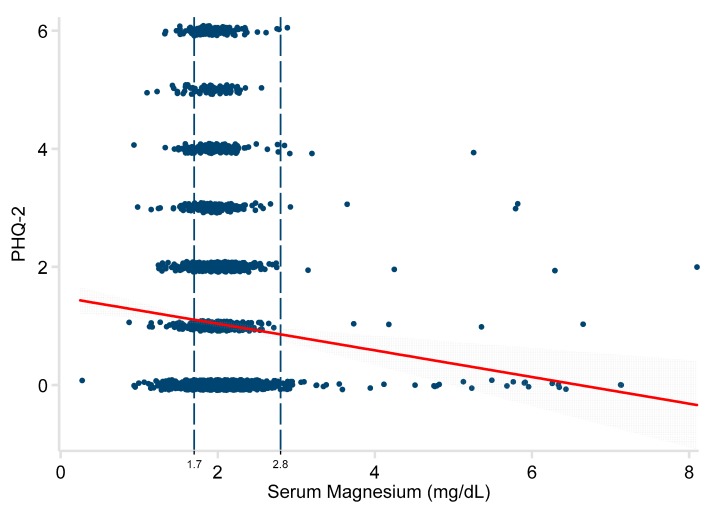
Relationship of serum magnesium to PHQ-2 scores in 3596 adults. Patient Health Questionnaire-2 (PHQ-2). The red line represents the least-squares regression estimate. The grey area demonstrates the 95% confidence interval around that estimate. The vertical dashed lines represent the normal range of serum magnesium.

The relationship between the serum magnesium level and PHQ-2 was significant in univariate regression analysis (−0.19 points/mg/dL; 95% CI −0.31, −0.07; *P* = 0.003). See Figure 2. After adjusting for age, the association between magnesium and PHQ-2 score was even stronger (−0.27 points/mg/dL; 95% CI −0.39, −0.14; *P* < 0.001). Further adjustment for sex, time between tests, race, diabetes, and chronic kidney disease had little additional effect (−0.23 points/mg/dL; 95% CI −0.35, −0.11 *P* = 0.001). See Table 2. Repeating the analysis using only the 3232 subjects with a normal serum magnesium level (1.7 to 2.8 mg/dL) showed an even stronger association (−0.43 points/mg/dL; 95% CI −0.70, −0.15; *P* = 0.002), even when controlling for all potential confounders. 

**Figure 3:**
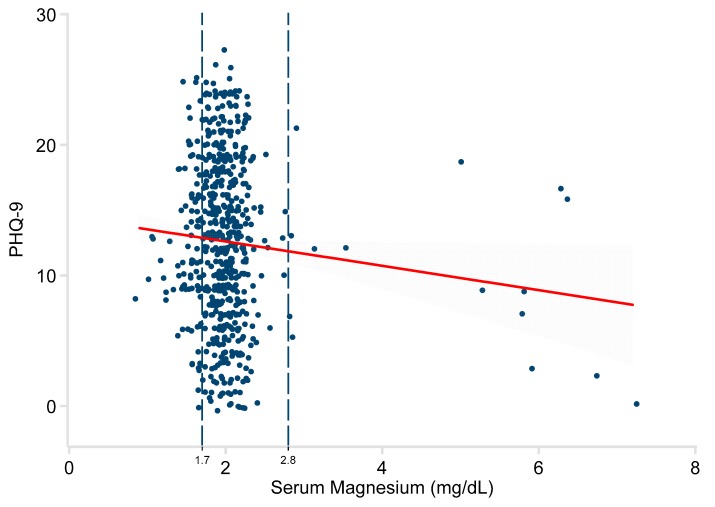
Relationship of serum magnesium to PHQ-9 scores in 616 adults. Patent Health Questionnaire-9 (PHQ-9). The red line represents the least-squares regression estimate. The grey area demonstrates the 95% confidence interval around that estimate. The vertical dashed lines represent the normal range of serum magnesium.

A similar relationship was observed for the PHQ-9 responses. The univariate model showed a significant effect (−0.93 points/mg/dL; 95% CI −1.81, −0.06; *P* = 0.037). See Figure 3. The relationship became stronger after adjustment. See Table 2. The results for all analyses were essentially identical, using robust regression methods, and are not shown here.

## 4. Discussion

The prevalence of depression is increasing. However, over 30% of patients with depressive symptoms do not receive treatment [7,29,30]. Reasons patients do not pursue treatment include cost and side effects of antidepressant medications, fear of perceived risk of addiction and dependence, and stigma associated with a mental health treatment [31,32]. In this population of adults receiving primary care, each additional mg/dL of serum magnesium was associated with about one quarter fewer points on the PHQ-2 depression scale after adjusting for age, gender, race, diabetes, kidney disease, and the time between measurement of serum magnesium and PHQ-2. Likewise, each additional mg/dL of serum magnesium was associated with about one less point on the PHQ-9 depression scale after adjusting for the same variables. 

The mechanism of the association between magnesium and depression is unknown. Magnesium plays a role in many of the pathways, enzymes, hormones, and neurotransmitters involved in mood regulation [33]. Similar to traditional antidepressant treatment, magnesium supplementation increases production of the brain-derived neurotropic factor (BDNF) [34]. The effect of magnesium on BDNF may be connected to magnesium’s role as a calcium antagonist and voltage-dependent blocker of the *N*-methyl-D-aspartate (NMDA) channel. NMDA regulates the flow of calcium into the neuron [35]. In low magnesium states, high levels of calcium and glutamate may deregulate synaptic function, resulting in depression [36]. Depression and magnesium are also both associated with systemic inflammation [37,38]. Increased cellular calcium during magnesium deficiency may be the primary mechanism through which magnesium status influences inflammation [39].

Treatment with oral magnesium, perhaps via dietary supplements, may be more appealing to many patients than other currently available approaches. Supplementation with magnesium leads to symptom improvement in depression [9,17] and increases serum magnesium levels in some studies [10,40], but not others [41,42]. A significant relationship between serum magnesium levels and depression has been reported in Iranian (*P* = 0.02; [43]) and Canadian (*P* < 0.04; [44]) populations. A recent meta-analysis of observational studies found an overall 1.3-fold increased risk of depression in people with hypomagnesaemia, but results were marginally insignificant after a sensitivity analysis [45]. Previous meta-analyses were inconclusive [24,46]. One study found that higher plasma magnesium levels at baseline led to a greater response to antidepressant treatment (OR = 32.5; 95% CI 1.1, 957.2; *P* = 0.04) [23]. Magnesium is listed as a short fall nutrient in the 2015–2020 Dietary Guidelines for Americans [47], and encouraging increased consumption of high magnesium foods is advisable, but may not lead to the same outcomes seen with supplementation. Identifying total body magnesium deficiency is difficult due to the limitations of serum magnesium levels [48,49]. During times of low magnesium intake, the response of the body is to increase absorption from dietary sources, reabsorption by the kidneys, and resorption from the bones (the major reserve of magnesium in the body) to maintain homeostasis. 

In the present study, the association between serum magnesium levels and depression was seen even when magnesium was in the normal range In other words, hypomagnesemia was not a prerequisite for the relationship. Many clinical trials that report a positive effect of magnesium on depression symptoms included subjects with known hypomagnesaemia [10,50]. However, those that did not limit the included population to people with hypomagnesaemia also found a positive effect [9,41]. The relationship between the baseline magnesium status and treatment outcome with magnesium supplementation needs further exploration. 

The prevalence of depression is increasing, representing an important public health problem [3]. The treatment method that ultimately leads to an acceptable level of improvement in depressive symptoms for any individual is unpredictable. There is currently a lack of biological measures to predict the efficacy of interventions and identify patients who will experience treatment resistance to traditional interventions. Identifying a biological predictor that can identify treatment resistance would lead to safer, faster, and more effective therapy, improved treatment recommendations, and reduced suffering [51]. Results from this study indicate serum magnesium levels may be a cost-effective means of identifying patients who may benefit from magnesium. Alternatively, serum magnesium may act as a marker for treatment-resistant depression [52], or perhaps identify altered magnesium metabolisms in a subgroup of patients who have a different treatment response than expected [23]. 

This study has several strengths including the large sample size recruited from a clinically relevant population. We were able to show the association of serum magnesium levels with two measures of depression (the PHQ-2 and PHQ-9), both of which are frequently used in the primary care population. We also acknowledge several limitations. This secondary data analysis can only show an association between serum magnesium levels and symptoms of depression. Although confounding by several important factors was included in the analysis, unmeasured confounders may be present. In particular, both depression and serum magnesium are associated with some chronic diseases. Therefore, the associations observed between serum magnesium and depression may be the result of confounding by the presence of chronic disease. The analysis controls for two of the most likely such confounders (diabetes and chronic kidney disease), but cannot exclude additional unmeasured confounding. The fact that magnesium levels were obtained suggests that the population may be sicker than the general adult population, limiting generalizability. Although Vermont is similar to other rural parts of the country, the results may not generalize more broadly. 

## 5. Conclusions

In this sample of adults receiving primary care, lower serum magnesium levels were associated with greater depressive symptoms, suggesting a biologic mechanism for the efficacy of magnesium supplementation to treat depression. Obtaining serum magnesium levels is safe, relatively easy, and inexpensive and could help individualize treatment.

## Figures and Tables

**Table 1:** Subject characteristics.

	PHQ-2		PHQ-9	
	(*n* = 3596)		(*n* = 616) ^1^	
Characteristic	Mean	Range	Mean	Range
Age (years)	62.0	18–99	56.1	18–99
Male	41.9%		38.8%	
White Race	94.6%		93.8%	
Diabetes	22.2%		24.0%	
Chronic Kidney Disease	8.2%		8.6%	
Months Between PHQ and Mg Test	7.4	0–35.1	8.1	0–34.4
Serum Mg (mg/dL) ^2^	2.0	<0.5–8.1	2.0	0.9–7.2
PHQ Score	1.0	0–6	12.6	0–27
0–2 (“Negative screen”)	84.8%		--	
3–6 (“Positive screen”)	15.2%		--	
0–4 (“None”)	--		11.2%	
5–9 (“Mild”)	--		22.2%	
10–14 (“Moderate”)	--		27.4%	
15–19 (“Severe”)	--		24.2%	
20–27 (“Very severe”)	--		14.9%	
≥10 (“Positive”)			66.5%	

Patient Health Questionnaire (PHQ); magnesium (Mg); ^1^ 608 subjects had both PHQ-2 and PHQ-9 results and appear in both analyses. ^2^ Normal serum magnesium levels = 1.7 to 2.8 mg/dL.

**Table 2:** Multivariate regression analyses.

	PHQ-2			PHQ-9		
	(*n* = 3590)			(*n* = 616) ^1^		
	*β*	*95% CI*	*P*	*β*	*95% CI*	*P*
Serum Mg (mg/dL)	−0.23	−0.35, −0.11	<0.001	−1.09	−1.96, −0.21	0.015
Age (year)	−0.02	−0.02, −0.01	<0.001	−0.05	−0.08, −0.02	0.001
Male	−0.19	−0.30, −0.09	<0.001	−0.78	−1.79, 0.23	0.13
Months Between PHQ and Mg	−0.01	−0.02, −0.00	0.011	−0.08	−0.14, −0.01	0.016
White	0.01	−0.22, 0.25	0.90	1.21	−0.82, 3.24	0.24
Diabetes	0.21	0.08, 0.34	0.001	0.74	−0.46, 1.93	0.23
Chronic Kidney Disease	0.35	0.15, 0.54	0.001	−1.10	−2.92, 0.72	0.24
Constant (intercept)	2.63	2.22, 3.03	<0.001	17.19	13.96, 20.41	<0.001

Magnesium (Mg); Patent Health Questionnaire (PHQ). ^1^ 608 subjects had both PHQ-2 and PHQ-9 results and appear in both analyses. *β =* the independent regression coefficient when controlling for all the other variables in the model. Confidence interval (CI).
